# Energy-Efficient Automated Detection of OPGW Features for Sustainable UAV-Based Inspection

**DOI:** 10.3390/s26020658

**Published:** 2026-01-19

**Authors:** Xiaoling Yan, Wuxing Mao, Xiao Li, Ruiming Huang, Chi Ye, Faguang Li, Zheyu Fan

**Affiliations:** 1 Guangzhou Electric Power Design Institute, Guangzhou 510630, China; 2 School of Intelligent Systems Engineering, Shenzhen Campus of Sun Yat-Sen University, Shenzhen 518107, China

**Keywords:** UAV-based inspection, OPGW, small object detection, YOLO11

## Abstract

Unmanned Aerial Vehicle (UAV)-based inspection is crucial for the maintenance and monitoring of high-voltage transmission lines, but detecting small objects in inspection images presents significant challenges, especially under complex backgrounds and varying lighting. These challenges are particularly evident when detecting the wire features of optical fiber composite overhead ground wire and conventional ground wires. Optical fiber composite overhead ground wire (OPGW) is a specialized cable designed to replace conventional shield wires on power utility towers. It contains one or more optical fibers housed in a protective tube, surrounded by layers of aluminum-clad steel and/or aluminum alloy wires, ensuring robust mechanical strength for grounding and high-bandwidth capabilities for remote sensing and control. Existing detection methods often struggle with low accuracy, insufficient performance, and high computational demands when dealing with small objects. To address these issues, this paper proposes an energy-efficient OPGW feature detection model for UAV-based inspection. The model incorporates a Feature Enhancement Module (FEM) to replace the C3K2 module in the sixth layer of the YOLO11 backbone, improving multi-scale feature extraction. A P2 shallow detection head is added to enhance the perception of small and edge features. Additionally, the traditional Intersection over Union (IoU) loss is replaced with Normalized Wasserstein Distance (NWD) loss function, which improves boundary regression accuracy for small objects. Experimental results show that the proposed method achieves a ${\mathrm{mAP}}_{50}$ of 78.3% and ${\mathrm{mAP}}_{50–95}$ of 52.0%, surpassing the baseline by 2.3% and 1.1%, respectively. The proposed model offers the advantages of high detection accuracy and low computational resource requirements, providing a practical solution for sustainable UAV-based inspections.

## 1. Introduction

With the advancement of technology and the modernization of power systems, traditional methods of power line inspection have gradually been replaced by more efficient and precise Unmanned Aerial Vehicle (UAV)-based inspections [1,2]. UAV-based inspections utilize high-definition cameras, infrared thermography cameras, and other sensors to monitor and collect data on power lines and related equipment in real-time [3]. Compared to traditional manual inspections, UAV-based inspections offer several advantages. First, UAVs can reach high altitudes or hazardous areas that are difficult for humans to access, reducing safety risks in the inspection process [4]. Second, UAV-based inspections are highly efficient and precise, enabling coverage of a large area of power infrastructure and completing tasks in a relatively short time. Most importantly, the various sensors mounted on UAVs provide real-time data on the status of electrical equipment, significantly enhancing the intelligence level of operation and maintenance in power systems. As a result, UAV-based inspections have become an indispensable technological tool in the power industry, widely applied in the inspection of power transmission lines, equipment maintenance, and fault diagnosis, among other areas.

**Figure 1:**
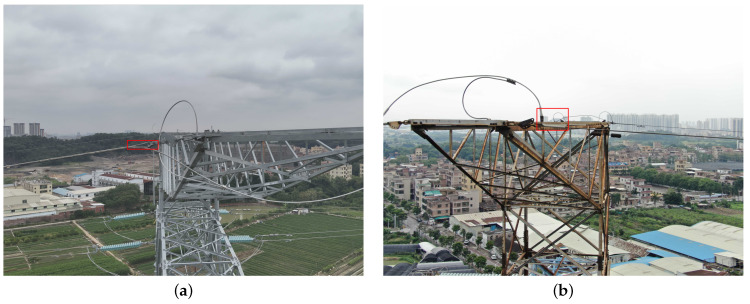

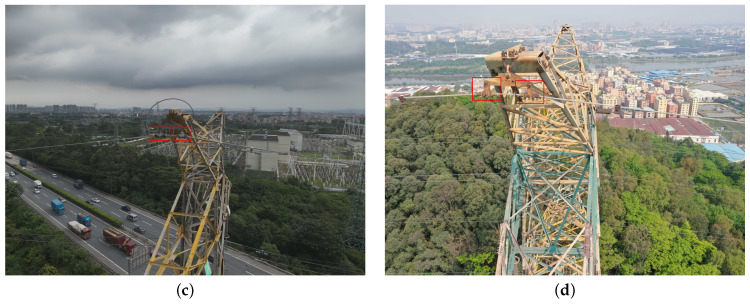
The four wire feature types of tension tower and tangent tower. (**a**) O1: wire feature of OPGW on tension tower. (**b**) D1: wire feature of conventional ground wire on tension tower. (**c**) O2: wire feature of OPGW on tangent tower. (**d**) D2: wire feature of conventional ground wire on tangent tower.

In the process of UAV-based inspections, the image data collected needs to be analyzed and interpreted through object detection technology to enable automatic identification and fault location of power equipment [5]. Image-based object detection plays a crucial role in UAV-based inspections, especially for power line inspection tasks. Specifically, we focus on how to utilize object detection technology to accurately detect OPGW (optical fiber composite overhead ground wire) and conventional ground wires in inspection images. OPGW, a key component of modern power transmission systems, serves the dual functions of lightning protection and optical fiber communication, which greatly enhance the stability and intelligence of power systems. Compared to conventional ground wire, OPGW exhibits significant differences in shape, thickness, and material properties. Particularly, on different types of towers (such as tension towers and tangent towers), OPGW and conventional ground wire show distinct wire features. As shown in Figure 1, the wire features of OPGW and conventional ground wires differ on tension towers and tangent towers, which can be classified into four categories. On tension towers, the wire feature of OPGW is a heart-shaped loop, labeled as O1, while the wire feature of the conventional tension tower ground wire is a tension clamp, labeled as D1. On tangent towers, OPGW typically uses preformed suspension clamps as its wire feature, labeled as O2, while the conventional tangent tower ground wire is characterized by a traditional boat-shaped suspension clamp, labeled as D2. By employing object detection technology to accurately recognize and distinguish these wire features in UAV-based inspection images, it is possible to effectively differentiate between OPGW and conventional ground wires, providing reliable data support for power line inspection and maintenance.

Image-based object detection technology plays a crucial role in UAV-based inspections, but challenges persist in its practical use, particularly when it comes to detecting OPGW and conventional ground wires. One major issue is that the wire features of OPGW and conventional ground wires often appear as small objects in images. This can lead to blurring or a loss of detail when captured from a distance, making them difficult to detect. Additionally, power lines are commonly situated in complex environments, where factors like lighting variations, cluttered backgrounds, and overlapping cables further complicate detection. Under different weather or lighting conditions, the wire features of OPGW and conventional ground wires can become tiny and low contrast, rendering traditional image processing methods inefficient and unreliable. Moreover, in cases of limited image resolution, object overlap, occlusion, or partial damage, detection algorithms may fail to identify or misidentify the wires. To enhance detection accuracy and robustness in real-world applications, it is crucial to consider these factors and optimize image processing algorithms and models, ensuring they can effectively recognize OPGW and conventional ground wires in challenging environments.

In recent years, with the rapid development of deep learning technology, object detection algorithms have been widely applied in power equipment inspection, fault detection, and image recognition. Among the various object detection algorithms, the YOLO (You Only Look Once) series has become one of the preferred methods in power equipment inspection due to its high real-time performance, low computational resource requirements, and excellent accuracy. The advantages of the YOLO series lies in its ability to quickly and accurately perform object recognition and localization tasks with minimal computational resources, making it particularly advantageous in scenarios such as power equipment inspection, where real-time performance is crucial. However, the traditional YOLO architecture still faces some significant limitations when dealing with the wire features of OPGW and conventional ground wires, which are small objects. These limitations manifest in several ways: first, the YOLO algorithm suffers from insufficient feature representation when handling small objects, resulting in the ineffective extraction of features for small-sized wires. Second, YOLO’s sensitivity to small objects is limited, especially in complex backgrounds, where the object can easily be confused with the background, leading to a decrease in recognition accuracy. Lastly, YOLO also has certain shortcomings in bounding box regression accuracy, particularly in complex scenes, where the localization error of the bounding boxes tends to be larger.

To address the aforementioned issues, this paper proposes an improved YOLO11-based model for detecting wire features of OPGW and conventional ground wires. The objective is to enhance the model’s detection accuracy and robustness in complex backgrounds through a series of innovative designs. Specifically, the main contributions of this paper include the following aspects:

(1) Feature Enhancement Module (FEM): In the backbone network of YOLO11, the original C3K2 module in the sixth layer is replaced by the FEM module. This module enhances the multi-scale feature extraction capability, effectively improving the representation of small structures, particularly for the feature extraction and representation of small objects such as wire feature. As a result, the detection accuracy is significantly improved.
(2) Introduction of Shallow Detection Head: A P2 shallow detection head is added to the Head section of the model to enhance its ability to perceive small-scale objects and fine details at the edges. By focusing on features from shallower layers, the P2 detection head effectively improves the model’s ability to recognize small objects (such as wire features of OPGW and conventional ground wires), overcoming the limitations of traditional YOLO architecture in small-object detection.
(3) Improvement of Loss Function: In the traditional YOLO model, the detection head uses the Intersection over Union (IoU) loss function. However, in complex backgrounds, the IoU loss function has limited performance in bounding box regression. To address this, this paper replaces the IoU loss function with the Normalized Wasserstein Distance (NWD) loss function. The NWD loss function provides higher accuracy and stability when handling bounding box regression in complex scenes, effectively enhancing the model’s performance in challenging environments.
(4) Low computational resource consumption and high detection accuracy: The WFD-YOLO network inherits the advantages of YOLO, maintaining a relatively small model parameter size and low computational resource consumption. Compared with other object detection models, it performs better in detecting wire features, achieving a good balance between computational resource requirements and detection accuracy.

In the M-scale model experiments, the results indicate that under consistent experimental conditions, the proposed method significantly improved detection accuracy. Specifically, the mean Average Precision at a single IoU threshold of 0.50 (${\mathrm{mAP}}_{50}$) reached 78.3%, an improvement of 2.3%, while the mean Average Precision across IoU thresholds uniformly sampled from 0.50 to 0.95 with a step size of 0.05 (${\mathrm{mAP}}_{50–95}$) achieved 52.0%, an increase of 1.1%. In addition, the method proposed in this paper inherits the lightweight characteristics of the YOLO network, has relatively low model parameters, and requires less computational power compared to other object detection models. These experimental results indicate that the proposed improved method significantly outperforms the traditional YOLO11 model in terms of detection accuracy, generalization ability, and capability to adapt to complex scenarios. Moreover, it has the advantage of low computational resource requirements, making it better suited for OPGW and conventional ground wire inspection tasks in complex environments during UAV-based inspections.

Overall, in response to the stringent energy efficiency requirements for UAV-based inspection scenarios, the WFD-YOLO network achieves ultra-low energy consumption while ensuring high-precision detection of wire features. By optimizing the model architecture, the minimized model parameters of WFD-YOLO effectively reduce the consumption of onboard computing resources. This improvement in computational efficiency enables WFD-YOLO to achieve an ideal balance between detection accuracy and energy consumption, providing a practical solution for sustainable UAV-based inspections.

## 2. Related Work

### 2.1. General Object Detection Algorithms

Object detection is a prerequisite for analyzing UAV-captured imagery. Algorithms are generally categorized into two paradigms: two-stage architectures (e.g., Faster R-CNN [6]) and one-stage architectures (e.g., SSD [7], YOLO series [8,9,10,11,12]). While Transformer-based methods (e.g., Swin Transformer [13]) offer strong global modeling capabilities, they often incur high computational costs that are unsuitable for the limited resources of airborne edge platforms. Conversely, the YOLO (You Only Look Once) series has become the industry standard for real-time applications due to its balance of speed and accuracy. Specifically, the latest iterations, such as YOLOv8 [9] and YOLO11 [12], have introduced efficient components like anchor-free heads and C2PSA blocks, significantly reducing parameter counts while maintaining high precision. Consequently, this study utilizes the lightweight YOLO architecture as a baseline to satisfy the stringent real-time requirements of UAV-based inspection.

### 2.2. Object Detection in Power Line Inspection

Detecting small objects such as wire features of OPGW and transmission lines presents unique challenges compared to general object detection. Early approaches relied on traditional edge detection and Hough transforms, which often failed in cluttered backgrounds or required extensive manual parameter tuning [14]. With the advent of deep learning, researchers have tailored CNNs specifically for transmission line components. For example, Rong et al. [15] improved wire detection by integrating directional filters and Oriented Bounding Boxes (OBBs) into the YOLO architecture to better capture the linear features of power lines against complex vegetation. Similarly, Barros et al. [16] utilized a multi-modal approach combining visual and thermographic data to enhance the robustness of power line detection in varying conditions. More recently, Yuan et al. [17] applied the latest YOLO11 architecture with spatial-channel dynamic inference to detect foreign objects on transmission lines, effectively balancing model size with the need for high-precision detection of small objects. Despite these advancements, standard detectors still struggle to distinguish small OPGW features when the wire diameter occupies only a few pixels in the UAV’s field of view.

### 2.3. Robustness in Complex Environmental Conditions

UAV-based inspection of OPGW is frequently compromised by environmental factors such as backlight, fog, and complex terrain backgrounds. To address this, recent research has focused on enhancing detection robustness. Guo et al. [18] proposed Zero-DCE to enhance image details under low-light conditions, while Liu et al. [19] introduced IA-YOLO, an adaptive framework for adverse weather. Specific to inspection scenarios, Guo et al. [20] optimized YOLOv7 with InceptionNeXt modules to handle vehicle detection in severe weather, and Liu et al. [21] integrated Receptive Field Blocks (RFBs) to improve accuracy in foggy conditions. Additionally, Ogino et al. [22] developed domain-specific filtering for high-precision detection under harsh lighting.

However, a critical gap remains: most existing improvements for adverse conditions focus on larger objects (e.g., vehicles, insulators) rather than the small wire features of OPGW and conventional groud wires. Furthermore, many optimized algorithms achieve robustness by significantly increasing model complexity. This structural redundancy leads to high energy consumption, which is incompatible with the endurance and thermal constraints of UAV edge computing platforms. Therefore, there is an urgent need for a model that concurrently addresses small OPGW feature extraction, environmental robustness, and extreme computational efficiency.

## 3. Methods

### 3.1. WFD-YOLO Model Overview

**Figure 2:**
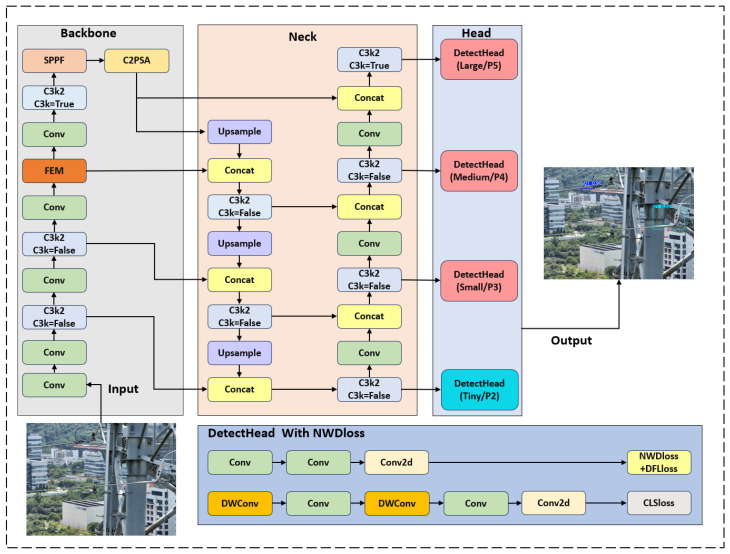
Model architecture of WFD-YOLO.

The WFD-YOLO network model we proposed uses YOLO11 as the baseline and is designed for fast and accurate detection of small wire features in OPGW and conventional ground wire images. By optimizing and improving the backbone network, loss function, and detection head of the YOLO11 model, we are able to significantly enhance the recognition accuracy and robustness for small objects, particularly under complex backgrounds and adverse lighting conditions. The overall architecture of the WFD-YOLO model, as shown in Figure 2, ensures significantly improved performance in detecting small wire features while maintaining a relatively small number of model parameters and low computational resource requirements.

First, to address the limitations of the traditional YOLO11 backbone network in handling small objects, we replaced the C3K2 module in the 6th layer with the Feature Enhancement Module (FEM). The FEM adopts a multi-branch convolution structure and dilated convolutions, which effectively enhance small-object feature extraction capabilities without significantly increasing the computational load. This enables the model to extract features from richer local information, improving the representation of small objects. The introduction of the FEM allows the network to capture the features of small-sized objects such as wire features of OPGW and conventional ground wires in greater detail, avoiding the problem of inaccurate small-object detection due to insufficient feature representation in the traditional YOLO model.

Secondly, to further improve the accuracy and fine detail perception of small object detection, we introduced an additional shallow object detection head based on the P2 feature map, built on top of the three-scale detection heads in YOLO11. The P2 detection head focuses on processing relatively shallow feature maps and is better at capturing local features of small objects, addressing the issue of feature loss for small objects in deep feature maps due to the excessive depth of convolutional layers. This structural improvement allows the WFD-YOLO model to significantly enhance detection accuracy when dealing with small objects, particularly in the edges and details of the object, resulting in more precise detection outcomes.

Moreover, to further enhance the model’s performance in complex scenarios, we improved the loss function. The traditional YOLO model uses an Intersection over Union (IoU)-based loss function, which can be influenced by bounding box regression errors, especially when there is low distinguishability between the object and the background. Therefore, we replaced the traditional IoU loss function with the Normalized Wasserstein Distance (NWD) loss function. The NWD loss function is more accurate in measuring the distance between the object and the background, particularly in complex backgrounds, effectively reducing bounding box regression errors and improving the accuracy and robustness of small-object detection. The introduction of NWD makes the WFD-YOLO model more stable in complex environments, enabling it to adaptively adjust to various scenarios and avoid detection failures or false positives that are common in traditional methods under complex backgrounds.

### 3.2. Feature Enhancement Module

The backbone network, as the fundamental feature extraction module of YOLO11, adopts a lightweight design concept aimed at reducing computational complexity while maintaining powerful feature extraction capabilities. In the tasks of detecting small wire features in images of OPGW and conventional ground wires, the backbone network, in the early stage of feature extraction, faces challenges due to its limited receptive field and insufficient feature richness. This makes it difficult to accurately capture the critical information of small objects and effectively distinguish them from the background. Therefore, a targeted Feature Enhancement Module needs to be designed to overcome these problems.

**Figure 3:**
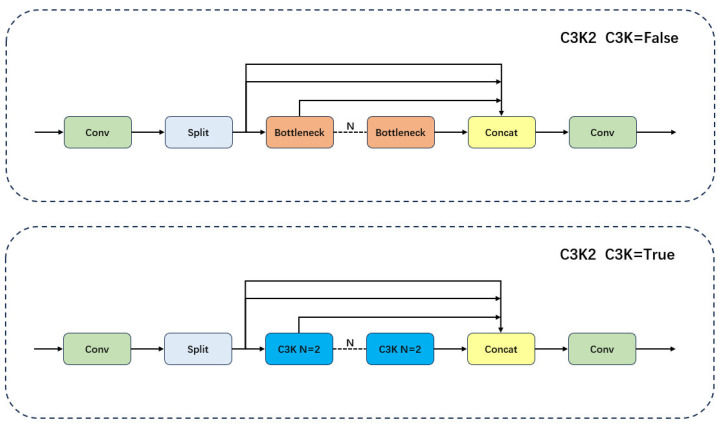
Structure of the C3K2 module.

**Figure 4:**
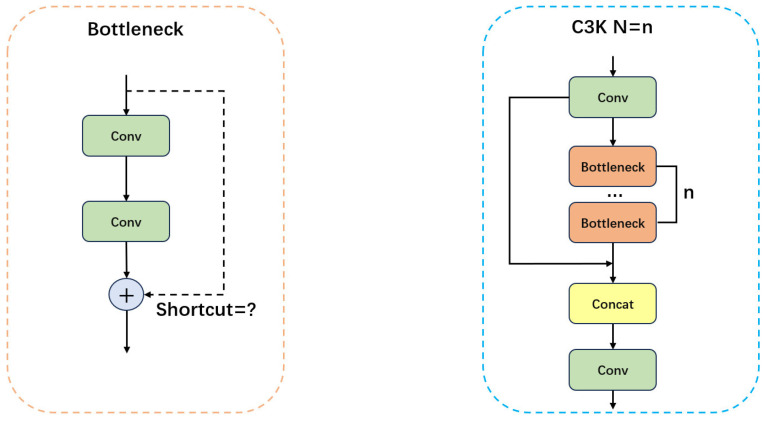
Structure of the C3K and Bottleneck modules.

In the traditional YOLO11 backbone network, key features are extracted by progressively downsampling the input image layer by layer, and then transmitted to the neck part via the C3K2 module at layer 4 and 6, as well as the C2PSA module at layer 10. Among these, the key features of small objects are mainly transmitted by the C3K2 module located at layer 6, the structure of which is shown in Figure 3. When the C3k parameter is set to True, the intermediate operation of the C3K2 module involves the parallel concatenation of feature maps from multiple C3K modules. When the C3k parameter is False, the intermediate operation consists of the parallel concatenation of feature maps from multiple Bottleneck modules, with the structures of the C3K and Bottleneck modules shown in Figure 4.

In the wire features detection tasks, the C3K2 module enhances feature richness by parallel concatenating convolutional feature maps of different sizes. However, its ability to capture local contextual information is limited, especially when dealing with the similarity between dense backgrounds and small objects, where the C3K2 module may struggle to effectively distinguish between the object and the background. Moreover, although the C3K2 module strengthens feature representation through the parallel concatenation of multiple feature maps, its relatively small convolution kernels and the lack of dilated convolutions result in a smaller receptive field when processing small objects. This implies that the C3K2 module cannot effectively expand the receptive field to capture global information of small objects, leading to significant limitations when dealing with small-object detection.

**Figure 5:**
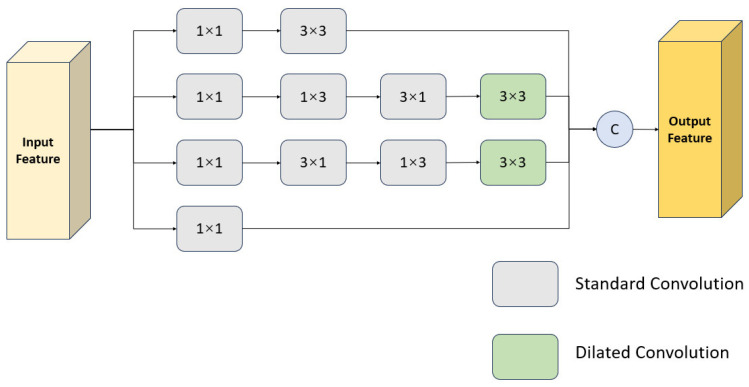
Structure of the Feature Enhancement Module.

To address the aforementioned issues, we introduce the Feature Enhancement Module (FEM) [23], which incorporates dilated convolution and multi-branch convolutional structures. By focusing on the two core dimensions of increasing feature richness and expanding the receptive field, the module accurately captures the critical information of small objects and effectively distinguishes them from the background. Its network structure is shown in Figure 5, which is primarily composed of the following four branches:

(1) $$W_{1}=f_{\mathrm{conv}}^{3\times 3}\left[f_{\mathrm{conv}}^{1\times 1}\left(F\right)\right],$$

(2) $$W_{2}=f_{\mathrm{diconv}}^{3\times 3}\left\{f_{\mathrm{conv}}^{3\times 1}\left[f_{\mathrm{conv}}^{1\times 3}\left[f_{\mathrm{conv}}^{1\times 1}\left(F\right)\right]\right]\right\},$$

(3) $$W_{3}=f_{\mathrm{diconv}}^{3\times 3}\left\{f_{\mathrm{conv}}^{1\times 3}\left[f_{\mathrm{conv}}^{3\times 1}\left[f_{\mathrm{conv}}^{1\times 1}\left(F\right)\right]\right]\right\},$$

(4) $$W_{4}=f_{\mathrm{conv}}^{1\times 1}\left(F\right),$$

where $f_{\mathrm{conv}}^{1\times 1}$, $f_{\mathrm{conv}}^{1\times 3}$, and $f_{\mathrm{conv}}^{3\times 3}$ represent the standard convolution operations with kernel sizes of 1×1, 1×3, and 3×3, respectively. $f_{\mathrm{diconv}}^{3\times 3}$ means dilated convolution operation with a dilation rate of 5. *F* is the input feature map. The final output feature map is obtained by combining the results of the four branches:

(5) $$Y=\mathrm{Cat}\left(W_{1},W_{2},W_{3}\right)⊕W_{4},$$

where Cat(·) is the feature map concatenation operation. ⊕ represents the elementwise addition operation of the feature map. $W_{1}$, $W_{2}$,$W_{3}$ and $W_{4}$ represent the output feature map of the four branches after standard and dilated convolution. *Y* is the output feature map of FEM.

### 3.3. P2 Shallow Detection Head

In the OPGW and conventional ground wire feature detection tasks, wire features are typically very small and easily obscured by background information, presenting a significant challenge for small-object detection. Since the wire features of OPGW and conventional ground wires generally have small sizes and low contrast, they are prone to being covered or confused with the surrounding background, especially in complex backgrounds or under significant lighting variations. Inspired by the work in [24], we introduced an additional P2 shallow detection head in the Head section of the YOLO11 model.

In the traditional YOLO11 model, the output layers typically include feature maps at P3, P4, and P5, which correspond to different scales of object detection capabilities. After three downsampling operations in the YOLO11 backbone network, feature maps of sizes 80 × 80 (P3), 40 × 40 (P4), and 20 × 20 (P5) are obtained. When processing an input image of size 640 × 640, YOLO11 gradually reduces the resolution of the feature maps through successive convolutional layers to extract features at various levels. However, each downsampling operation reduces the resolution of the feature maps, and the final P5 layer features a resolution of only 20 × 20. This means that as the network depth increases, the spatial resolution of the feature maps progressively decreases, and the model performs poorly when detecting very small objects, especially the heart-shaped ring feature in OPGW and the wire feature of conventional ground wire. This is because, as the number of convolutional layers increases, the features of small objects tend to become more blurred or even vanish in the higher-level feature maps.

In the feature maps from P3 to P5, the object detection capability gradually decreases, primarily due to the reduction in resolution of the feature maps, making it difficult for the model to capture fine features of small-scale objects. Assuming the input image has a size of H×W, the size $S_{k}$ of the feature map at each downsampling layer, after applying the convolutional operation, is given by the following formula:

(6) $$S_{k}=\frac{H\times W}{2^{k}},$$

where *H* is the height of the input image, *W* is the width of the input image, *k* represents the layer number, and $S_{k}$ is the size of the feature map obtained from the *k*-th layer.

For an input image with size 640×640, the feature map sizes after three downsampling operations are

(7) $$S_{3}=\frac{640\times 640}{2^{3}}=80\times 80,$$

(8) $$S_{4}=\frac{640\times 640}{2^{4}}=40\times 40,$$

(9) $$S_{5}=\frac{640\times 640}{2^{5}}=20\times 20.$$

It can be seen that as the size of the feature map decreases, the model’s ability to detect small objects drops significantly. This is because the traditional YOLO11 model in the OPGW and conventional ground wire features has certain spatial limitations, especially when the object has a complex background with more variations.

To address this issue, we introduced a new P2 detection head into the original YOLO11 model, adding a shallow convolutional layer (P2) that enables the model to focus more on smaller regions of the image. The feature map at layer P2 is much smaller than the feature maps at layers P3, P4, and P5, and it retains more object information, enabling more precise detection of objects with a finer scale. Specifically, P2 allows for more precise comparison of the feature map size between P2 and P3 layers, enabling the model to detect small objects more accurately, even when there are complex backgrounds or background noise.

Assuming the size of the feature map at layer P2 is $S_{2}$, we can express it as

(10) $$S_{2}=\frac{640\times 640}{2^{2}}=160\times 160.$$

Under this setting, the resolution of the P2 feature map is higher than that of P3 (80 × 80) and P4 (40 × 40), enabling it to retain more detailed information. This is particularly effective for handling small wire features of OPGW and conventional ground wires, significantly improving detection accuracy. By introducing the shallow feature map of the P2 layer into the detection head, the network can better utilize local image information, enhancing its ability to recognize small objects.

### 3.4. NWD Loss Function

The traditional YOLO model series uses the Intersection over Union (IoU) as the loss function, and its variants (e.g., GIoU, DIoU, CIoU) modify this loss.The specific formula is as follows:

(11) $$IoU=\frac{\left|A\cap B\right|}{\left|A\cup B\right|},$$

where *A* is the predicted bounding box and *B* represents the true bounding box.

However, this region-based Intersection over Union (IoU) metric has significant limitations in small-object scenarios. On the one hand, small objects occupy an extremely small proportion at the pixel level, and even slight positional shifts can cause a drastic decrease in IoU, potentially even to zero, resulting in vanishing gradients and unstable training. On the other hand, IoU only measures the overlap between the predicted and ground truth bounding boxes, lacking the ability to continuously model the deviations in the position and size of the object center, making it difficult to meet the fine localization requirements in tasks like the detection of wire features of small objects in OPGW and conventional ground wire images. To address this, we introduce the Normalized Gaussian Wasserstein Distance (NWD) [25] as the core loss function for bounding box regression, within the YOLO11 framework. NWD, from the perspective of probability distribution matching, models bounding boxes as two-dimensional Gaussian distributions. By calculating the Wasserstein distance between the predicted and ground truth distributions, NWD builds a continuous, differentiable, and position-sensitive optimization objective, fundamentally improving the accuracy and robustness of small-object detection. The NWD loss function is given by the following expression.

Let the predicted bounding box and the ground truth bounding box be represented as

(12) $$B_{p}=\left(x_{p},y_{p},w_{p},h_{p}\right),$$

(13) $$B_{t}=\left(x_{t},y_{t},w_{t},h_{t}\right).$$

Convert them into two-dimensional Gaussian distributions $N_{p}\left(\mu_{p},\Sigma_{p}\right)$ and $N_{t}\left(\mu_{t},\Sigma_{t}\right)$, defined as follows:

(14) $$\mu =\left[\begin{array}{c} x\\ y \end{array}\right],$$

(15) $$\Sigma =\left[\begin{array}{cc} \frac{w^{2}}{4} & 0\\ 0 & \frac{h^{2}}{4} \end{array}\right],$$

where the μ represents the center position of the bounding box, and the covariance matrix Σ reflects the width and height distribution, capturing the shape information of the bounding box.

Once $N_{p}$ and $N_{t}$ obtained by converting to two-dimensional Gaussian distributions, the second-order Wasserstein distance $W_{2}^{2}\left(N_{p},N_{t}\right)$ between the two Gaussian distributions can be calculated using the following formula:

(16) $$W_{2}^{2}\left(N_{p},N_{t}\right)={∥\mu_{p}-\mu_{t}∥}_{2}^{2}+{∥\Sigma_{p}^{1/2}-\Sigma_{t}^{1/2}∥}_{F}^{2},$$

where ${∥\cdot ∥}_{2}$ represents the Euclidean norm, and ${∥\cdot ∥}_{F}$ represents the Frobenius norm. These norms calculate the difference between the center position, width, and height in their respective dimensions.

However, the Wasserstein distance is inherently a distance metric, and its range is not within [0, 1], which is not conducive to loss normalization and network convergence. To tackle this, an exponential normalization strategy is introduced, mapping the Wasserstein distance to a similarity metric similar to IoU. The NWD is defined as follows:

(17) $$NWD\left(N_{p},N_{t}\right)=\exp\left(\frac{-\sqrt{W_{2}^{2}\left(N_{p},N_{t}\right)}}{c}\right),$$

where the constant *c* is used to control the scale invariance and is typically set to the empirical value of the average diagonal length of the object boxes in the dataset.

Finally, the loss function based on the NWD is defined as

(18) $$L_{NWD}=1-NWD\left(N_{p},N_{t}\right).$$

## 4. Experiments

### 4.1. Experimental Setup

#### 4.1.1. Dataset

We collected a total of 3137 wire images with multiple perspectives and complex backgrounds, each containing one or more wire features. The labeling process was carried out using the Labelimg annotation tool, resulting in the annotation of four categories of wire features. As shown in Figure 1, the four wire feature categories are O1, the OPGW feature of a tension tower; D1, the conventional ground wire feature of a tension tower; O2, the OPGW feature of a tangent tower; and D2, the conventional ground wire feature of a tangent tower. We selected 1882 wire images for the training set, 620 images for the validation set, and the remaining images were used for testing.

We present the distribution of object categories and object sizes in the dataset in Table 1 and Table 2, respectively. As shown in Table 1, the dataset maintains a relatively balanced distribution across categories, with the ratio between the largest category (O1) and the smallest category (D2) being 2.49:1 (2222:892). Specifically, the ratio of OPGW cable categories (O1 and O2) to traditional ground wire categories (D1 and D2) is almost equal, at 53.0% and 47.0%, respectively. Furthermore, the standard deviation of category distribution across the training, validation, and test sets is always less than 0.8%, indicating high consistency of data among different subsets. In addition, as shown in Table 2, 74.1% of the objects in the dataset occupy less than 0.5% of the image area, with an average of 0.49% per object, highlighting the small-scale nature of the detection objects. Only 3.8% are large objects, further emphasizing that our dataset focuses on typical challenging small-object detection scenarios.

#### 4.1.2. Implementation Details

All models were trained on an NVIDIA Quadro RTX 8000 (NVIDIA, Santa Clara, CA, USA). The WFD-YOLO model is based on YOLO11 and has three scales: N, S, and M. We implemented our method and trained it for 400 epochs using a training framework with a batch size of 8. The WFD-YOLO network was optimized using Stochastic Gradient Descent (SGD) [26], with a learning rate of 0.0001, momentum set to 0.937, and a weight decay of 0.0005. Input images were resized to 640 × 640 pixels, and the YOLO11 model’s data augmentation methods were used to ensure consistency across experiments. We report standard COCO metrics, including Precision, Recall, ${\mathrm{mAP}}_{50–95}$ (the mean Average Precision across IoU thresholds uniformly sampled from 0.50 to 0.95 with a step size of 0.05) and ${\mathrm{mAP}}_{50}$ (the mean Average Precision at a single IoU threshold of 0.50). In addition, we also reported the number of parameters (Params) and the number of floating-point operations per second (GFLOPs) to measure the computational and memory costs of the model, which are crucial for deployment on edge devices and energy consumption.

### 4.2. Comparative Experiments

**Figure 6:**
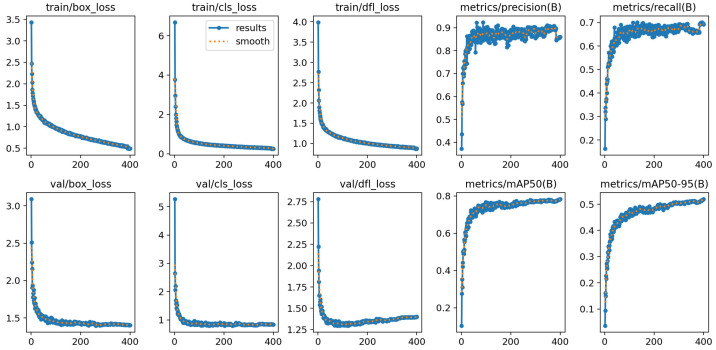
The variations of each parameter during the training process of WFD-YOLO-M.

As shown in Figure 6, the variation of various metrics during the training process of WFD-YOLO-M is presented, which we use as a representative of the training process for other scaled WFD-YOLO models. The final experimental results are summarized in Table 3. On the dataset of OPGW and conventional grounding wire features that we collected, our WFD-YOLO-N improved by 0.3% in ${\mathrm{mAP}}_{50–95}$ and increased by 0.9% in ${\mathrm{mAP}}_{50}$, outperforming the baseline YOLO11-N. Similarly, WFD-YOLO-S achieved a 1.1% increase in ${\mathrm{mAP}}_{50–95}$ and a 3.1% improvement in ${\mathrm{mAP}}_{50}$, surpassing the baseline. WFD-YOLO-M also demonstrated higher accuracy than YOLO11-M, with a 1.1% increase in ${\mathrm{mAP}}_{50–95}$ and a 2.3% rise in ${\mathrm{mAP}}_{50}$. Furthermore, we compared our method with other real-time object detectors in the YOLO series in terms of computational cost. The results show that our method, while outperforming other YOLO models of the same scale in terms of accuracy (Precision, Recall, and mAP), does not significantly increase in parameters or floating-point operations, and its computational demand remains advantageous within the real-time object detection model series.

As shown in Table 4, this table compares the differences in computational resource requirements and detection accuracy between WFD-YOLO and DETR-based models in the tasks of wire feature detection. The results indicate that WFD-YOLO inherits the lightweight characteristics of the YOLO network. Compared to DETR-based object detection models, it has significantly fewer parameters and floating-point operations, which means it requires less computational power. Despite the lower computational demands, it can still maintain excellent detection accuracy for wire features under these conditions.

### 4.3. Ablation Studies

We conducted ablation experiments using WFD-YOLO-S and WFD-YOLO-M on the OPGW and conventional ground wire feature dataset we collected, to analyze the impact of each component on detection accuracy. The performance comparisons of different configurations are shown in Table 5 and Table 6, where FEM represents the Feature Enhancement Module, P2Head refers to the newly added P2 shallow detection head branch, and NWD loss stands for the normalized Gaussian Wasserstein distance loss function.

In the S-scale experiment, the baseline model YOLO11-S achieved a ${\mathrm{mAP}}_{50}$ of 72.8 and a ${\mathrm{mAP}}_{50–95}$ of 46.9. After incorporating the Feature Enhancement Module, both metrics improved to 73.4 and 47.1, respectively, reflecting the significant enhancement effect of this module on image features. Further addition of the P2 shallow detection head led to a ${\mathrm{mAP}}_{50}$ of 74.0 and an increase in ${\mathrm{mAP}}_{50–95}$ to 47.2. While this addition incurred a computational cost increase of approximately 30% (from 23.4 to 30.4 GFLOPs), it effectively enhanced the extraction of finer object information. Ultimately, the fully integrated WFD-YOLO-S, which includes all modules, achieved the best performance with a ${\mathrm{mAP}}_{50}$ of 75.9 and a ${\mathrm{mAP}}_{50–95}$ of 48.0.

In the M-scale ablation study, the baseline model YOLO11-M achieved a ${\mathrm{mAP}}_{50}$ of 76.0 and a ${\mathrm{mAP}}_{50–95}$ of 50.9. After adding the FEM, the performance further improved to 76.2 and 51.1, validating the module’s ability to enhance the expression of image features. The introduction of the P2 shallow detection head raised the ${\mathrm{mAP}}_{50}$ to 76.9 and ${\mathrm{mAP}}_{50–95}$ to 51.5. Consistent with the S-scale results, the P2 head brought a significant GFLOPs increase (from 67.8 to 87.6) in exchange for improved detection on high-resolution features. Ultimately, the fully integrated WFD-YOLO-M, incorporating all modules, achieved a ${\mathrm{mAP}}_{50}$ of 78.3 and a ${\mathrm{mAP}}_{50–95}$ of 52.0, demonstrating that the cumulative effect of all the improved modules continuously drives performance enhancement in detection accuracy.

Cost–Benefit Analysis of the P2 Head: As observed in both Table 5 and Table 6, the inclusion of the P2 Head introduces a noticeable increase in GFLOPs (+30%) while the overall mAP improvement appears incremental (+0.6% to +0.7%). However, in the context of OPGW inspection within the IoT framework, this trade-off is strategically justified. The P2 Head preserves high-resolution spatial details essential for detecting minute defects and small wire deatures, which are the most challenging objects and often fatal if missed. The “marginal” mAP gain primarily reflects the recovery of these specific, hard-to-detect objects rather than a general accuracy boost. Furthermore, despite the higher GFLOPs, the computational demand of the S-model (30.4 GFLOPs) and even the M-model remains within the real-time processing envelope of mainstream industrial edge devices (e.g., NVIDIA Jetson Orin (NVIDIA, Santa Clara, CA, USA)). Thus, we prioritize high-reliability detection for safety-critical targets over minimizing purely theoretical computational costs.

### 4.4. Visualization

#### 4.4.1. Heatmap

**Figure 7:**
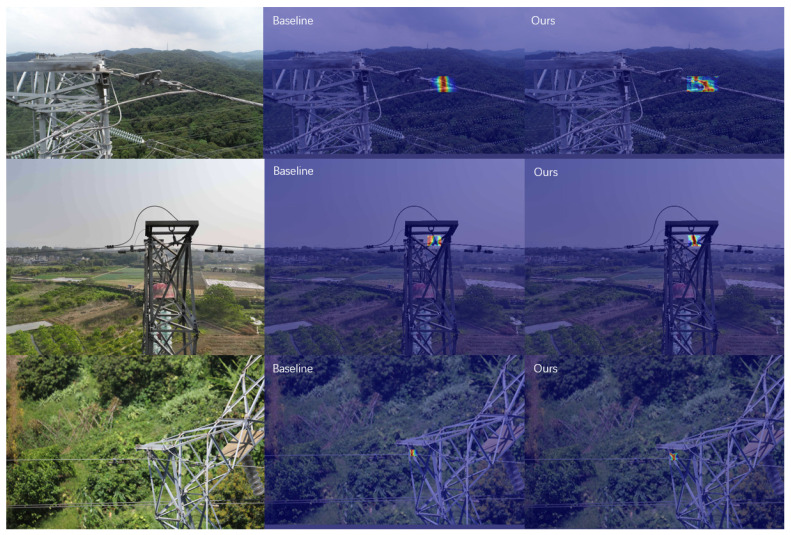
The visualization results of the M-scale model. Red colors indicate regions where the model focuses more attention.

As shown in Figure 7, we present a detailed heatmap comparison of WFD-YOLO-M and YOLO11-M in detecting wire features in OPGW and conventional ground wire images. Compared to the baseline model, WFD-YOLO shows a significant improvement in detecting wire features. From the image comparison in the second row, it can be seen that in the heatmap of the baseline model, the high-heat regions are mainly concentrated around the wire features, indicating that the model’s attention is not focused on the wire features themselves but is dispersed across the background information. In contrast, in the heatmap of our model, the high-heat regions closely match the shape of the wire features, suggesting that WFD-YOLO pays more attention to the wire features themselves rather than the background information. Additionally, from the comparison of the images in the first and third rows, it can be observed that, compared to the baseline model, WFD-YOLO has larger high-heat regions. This further validates that the WFD-YOLO model captures wire features more accurately. Therefore, WFD-YOLO demonstrates superior performance in detecting the wire features of OPGW and conventional ground wires.

#### 4.4.2. Comparison of Detection Results

**Figure 8:**
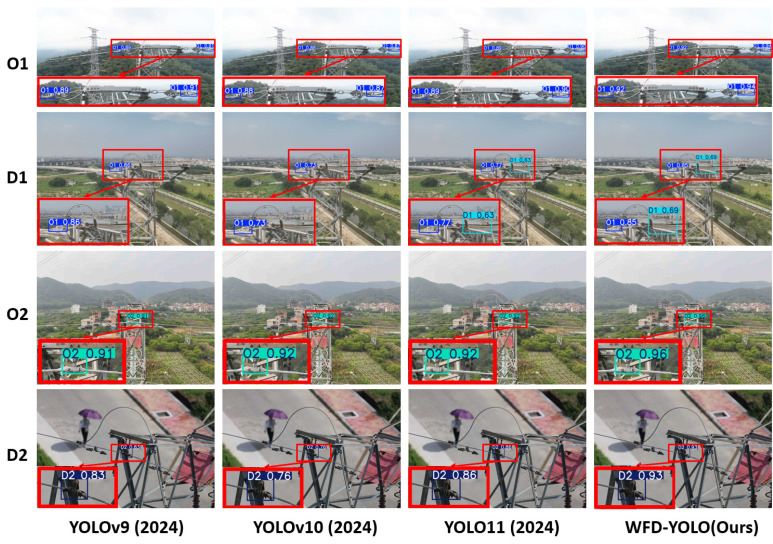
The visualization results of detection comparison.

As shown in Figure 8, we present a comparison of the detection results of YOLOv9, YOLOv10, YOLO11, and WFD-YOLO at M scale. The detection categories from top to bottom are O1, D1, O2, and D2. From the results shown in the figure, we can see that WFD-YOLO achieves higher detection accuracy than the other models for all four wire features. This fully demonstrates that WFD-YOLO has a significant advantage in the task of detecting wire features in drone inspection images. Compared to YOLOv9, YOLOv10, and YOLO11, WFD-YOLO is able to capture smaller wire feature edge details, and even in complex background environments (such as vegetation interference or low contrast), it still maintains very high localization accuracy and classification confidence.

## 5. Discussion

Based on experimental results, WFD-YOLO successfully improves the accuracy of small-object detection while maintaining low computational resource requirements through a series of innovative improvements within the YOLO framework.

First, the model introduces minimal computational overhead to enhance small-object features in images without increasing the complexity of model deployment. As shown in the experimental results, our model achieves higher accuracy in similar real-time detectors without a significant increase in computational demand. This improvement is closely linked to the Feature Enhancement Module in our model, which replaces the original C3K2 module. Compared to C3K2, FEM has relatively low complexity but effectively increases feature richness and expands the receptive field, thereby enhancing the feature representation of small wire features.

Second, our model improves the detection of small wire features that have been occluded. These wire features often occupy a small pixel area in images and can easily be overwhelmed by large-scale features on high-level feature maps. The additional detection head on the shallow feature maps captures more local information, significantly improving the detection accuracy of small wire features.

Finally, compared to other YOLO models, WFD-YOLO has more refined small-object detection, which enhances its ability to detect wire features. To further enhance bounding box regression accuracy, we replaced the original IoU-based loss function with NWD loss. Unlike traditional IoU-based losses, NWD loss measures the position and scale differences between the predicted and ground truth boxes from a spatial distribution perspective. This loss function is more capable of distinguishing subtle positional deviations, which is especially beneficial in small-object detection scenarios, where slight misalignments can lead to missed detections.

**Figure 9:**
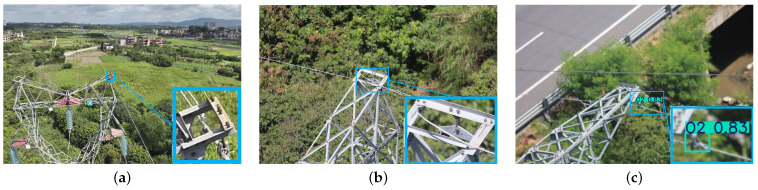
Some examples of detection failures. (**a**) Missed detection due to objects being too small. (**b**) Missed detection caused by occluded objects. (**c**) False detection caused by the image being too blurry. The actual feature in the image is D2, but it was identified as O2.

Despite these advancements, WFD-YOLO still faces certain limitations in extreme scenarios. As shown in Figure 9, when objects occupy an extremely small pixel area or are heavily occluded by complex environmental elements, the sparsity of available semantic information can lead to frequent missed detections. Furthermore, under conditions of severe image blur or low signal-to-noise ratios, the model may exhibit false positives, such as misidentifying background noise as wire features or failing to accurately distinguish between specific wire categories.

In summary, compared to other object detection models, WFD-YOLO inherits the advantages of the YOLO framework, maintaining a smaller number of parameters and lower computational resource requirements. Through our improvements, WFD-YOLO achieves higher detection accuracy in OPGW and conventional ground wire feature detection tasks, successfully balancing low computational power with high detection precision.

## 6. Conclusions

This paper proposes the WFD-YOLO network based on YOLO11 for efficiently detecting the wire features of OPGW and conventional ground wires, aiming to achieve high-precision detection with minimal computational resource consumption. By introducing the Feature Enhancement Module into the backbone network of YOLO11, adding the P2 shallow detection head in the detection head, and replacing the traditional IoU loss function with the Normalized Wasserstein Distance loss function, we effectively enhance the model’s ability to adapt to small-object wire features and complex scenarios. The FEM improves the feature representation of small objects by enhancing multi-scale feature extraction, while the P2 shallow detection head further improves detection capability by capturing more detailed local information. The NWD loss function provides higher accuracy and robustness in bounding box regression. Experimental results show that WFD-YOLO inherits the lightweight advantages of the YOLO series, with fewer parameters compared to other object detection models, thus consuming fewer computational resources. Under the same experimental conditions, compared to YOLO11, the ${\mathrm{mAP}}_{50}$ metric improved by 2.3%, reaching 78.3%, and the ${\mathrm{mAP}}_{50–95}$ metric increased by 1.1%, reaching 52.0%. These results demonstrate the significant advantages of the model in the tasks of detecting the wire features of OPGW and conventional ground wires. The proposed WFD-YOLO model provides an efficient and accurate solution for small-object detection, with broad application potential. In the future, the model can be further optimized for real-time performance and robustness, and extended to feature detection of other types of power transmission lines.

## Figures and Tables

**Table 1:** Dataset category distribution.

Category ID	Category Name	Training Set	Validation Set	Test Set	Total Instances	Overall Proportion
0	O1	1323 (34.9%)	448 (35.4%)	451 (35.0%)	2222	35.0%
1	D1	1251 (33.0%)	413 (32.6%)	423 (32.8%)	2087	32.9%
3	O2	681 (18.0%)	226 (17.8%)	236 (18.3%)	1143	18.0%
4	D2	533 (14.1%)	180 (14.2%)	179 (13.9%)	892	14.1%
Total	-	3788	1267	1289	6344	100%

**Table 2:** Relative object size distribution.

Relative Size Category	Area Proportion Range	Instance Count	Percentage	Characteristic Description
Tiny Objects	<0.1%	1582	24.9%	Near resolution limit, extremely difficult to detect
Small Objects	0.1∼0.5%	3124	49.2%	Primary detection objects
Medium Objects	0.5∼2.0%	1398	22.0%	Relatively easy to detect
Large Objects	>2.0%	240	3.8%	Close-range captures

**Table 3:** Comparison results between WFD-YOLO and other YOLO-series models. The bold values indicate the best performance.

Model	Input Size	Params (M)	GFLOPs	Precision	Recall	${\mathrm{mAP}}_{50–95}$	${\mathrm{mAP}}_{50}$
YOLOv9-T [10]	640 × 640	2.0	7.6	79.7	60.1	41.9	68.7
YOLOv9-S [10]	640 × 640	7.1	26.7	86.2	62.5	46.7	72.7
YOLOv9-M [10]	640 × 640	20.0	76.5	85.5	67.9	50.8	77.2
YOLOv10-N [11]	640 × 640	2.7	8.2	84.8	54.6	40.5	66.9
YOLOv10-S [11]	640 × 640	8.0	24.5	86.9	61.6	47.7	72.4
YOLOv10-M [11]	640 × 640	16.4	63.5	87.1	58.8	49.3	73.9
YOLO11-N [12]	640 × 640	2.6	6.3	84.1	68.3	41.8	69.8
YOLO11-S [12]	640 × 640	9.4	21.3	85.3	60.9	46.9	72.8
YOLO11-M [12]	640 × 640	20.0	67.7	86.9	63.7	50.9	76.0
WFD-YOLO-N (Ours)	640 × 640	3.7	11.4	85.1	61.7	42.1	70.7
WFD-YOLO-S (Ours)	640 × 640	11.0	30.4	89.4	66.4	48.0	75.9
WFD-YOLO-M (Ours)	640 × 640	20.6	87.6	**90.1**	**69.5**	**52.0**	**78.3**

**Table 4:** Comparison results of WFD-YOLO with DETR-based object detection models. The bold values indicate the best performance.

Model	Input Size	Params (M)	GFLOPs	Precision	Recall	${\mathrm{mAP}}_{50–95}$	${\mathrm{mAP}}_{50}$
RT-DETR-L [27]	640 × 640	32.0	103.5	**91.3**	67.8	44.8	72.4
RT-DETR-X [27]	640 × 640	65.5	222.5	89.5	72.1	46.3	74.1
UAV-DETR-EV2 [28]	640 × 640	13.2	44.0	86.8	70.6	45.3	73.2
UAV-DETR-R18 [28]	640 × 640	21.6	74.2	88.4	71.8	47.1	77.5
UAV-DETR-R50 [28]	640 × 640	45.5	166.4	89.7	**72.1**	48.6	77.8
WFD-YOLO-N (Ours)	640 × 640	3.7	11.4	85.1	61.7	42.1	70.7
WFD-YOLO-S (Ours)	640 × 640	11.0	30.4	89.4	66.4	48.0	75.9
WFD-YOLO-M (Ours)	640 × 640	20.6	87.6	90.1	69.5	**52.0**	**78.3**

**Table 5:** Ablation experiment results on WFD-YOLO-S.

Baseline	FEM	P2Head	NWDLoss	Params (M)	GFLOPs	${\mathrm{mAP}}_{50–95}$	${\mathrm{mAP}}_{50}$
✔				9.4	21.3	46.9	72.8
✔	✔			10.8	23.4	47.1	73.4
✔	✔	✔		11.0	30.4	47.2	74.0
✔	✔	✔	✔	11.0	30.4	48.0	75.9

**Table 6:** Ablation experiment results on WFD-YOLO-M.

Baseline	FEM	P2Head	NWDLoss	Params (M)	GFLOPs	${\mathrm{mAP}}_{50–95}$	${\mathrm{mAP}}_{50}$
✔				20.1	67.7	50.9	76.0
✔	✔			20.1	67.8	51.1	76.2
✔	✔	✔		20.6	87.6	51.5	76.9
✔	✔	✔	✔	20.6	87.6	52.0	78.3

## Data Availability

The data presented in this study are available on request from the corresponding author due to the data being part of an ongoing study.
